# Viscous Fingering and Dendritic Growth of Surface Crystallized Sr_2_TiSi_2_O_8_ Fresnoite

**DOI:** 10.1038/srep03558

**Published:** 2013-12-19

**Authors:** Wolfgang Wisniewski, Marek Patschger, Christian Rüssel

**Affiliations:** 1 Otto-Schott-Institut, Jena University, Fraunhoferstr. 6, 07743 Jena, Germany

## Abstract

During the quenching of a melt with the composition 2SrO**·**TiO_2_**·**2.75SiO_2_, cubic SrTiO_3_- and tetragonal Sr_2_TiSi_2_O_8_-crystals are formed at the surface. Subsequent crystal growth leads to dendritic fresnoite structures which become increasingly finer until the mechanism changes to viscous fingering during further cooling. In the final stages of this initial growth step, the crystal orientations of these dendrites systematically change. Due to a complete absence of bulk nucleation in this system, crystal growth is resumed upon reheating to 970°C and fractal growth with the c-axis tilted by about 45° from the main growth direction is observed. The results are interpreted to confirm the link between viscous fingering and dendritic growth in the case of a true crystallization process.

Recent work applying electron backscatter diffraction (EBSD) towards the surface crystallization of glass has provided surprising new results^1^,^2^,^3^,^4^,^5^,^6^,^7^,^8^. It was observed that the topmost crystals of some systems already show discrete orientation preferences in the information volume of EBSD. The latter has been stated to be less that 50 nm in depth^9^,^10^,^11^,^12^,^13^ although it has been proposed to be a bit larger, see discussion in Ref. 14. As none of the textures detected so far prevailed during growth into the bulk^1^,^2^,^3^,^4^,^5^ it is justified to conclude that oriented nucleation occurs in some glasses in contrast to the traditional assumption of statistically oriented nucleation. The latter also occurs, e.g. during the surface crystallization of cordierite^6^, yttrium aluminium garnet (YAG)^7^ and high-cristobalite^8^ from glasses.

Sr-fresnoite of the composition Sr_2_TiSi_2_O_8_ (STS) crystallized in a glass with some excess silica (Sr_2_TiSi_2_O_8_**·** 0.75SiO_2_) shows oriented nucleation with the [001]–direction perpendicular to the surface^5^. Subsequently a slow, continuous growth selection changes the texture towards the [001]-direction being tilted by about 45° from the surface normal after 300 μm of growth into the bulk. The additional SiO_2_ in the glass melt is added to allow comparability to the surface crystallization experiments of barium titanium silicate (BTS^1^) and STS^5^ and experiments concerning the electrochemically induced nucleation of fresnoite in glasses^15^,^16^,^17^,^18^,^19^. It was initially chosen to ease glass preparation and controlled crystallization as the nucleation tendency of the glass is reduced by the added SiO_2_. BTS has one of the highest nucleation rates known for glass-ceramics^20^ which makes it very difficult to produce a stoichiometric glass. It was shown that adding SiO_2_ to the stoichiometric BTS glass composition increases the viscosity at the respective temperature^21^. As the glass transition temperatures T_g_ of BTS (730°C) and STS (760°C) glasses with the same additional SiO_2_ are similar^1^,^5^, it is acceptable to assume the STS-glass viscosity will show a comparable dependency.

The system discussed in this article has raised questions towards the mechanism of STS-growth in glasses. While a model of reoccurring oriented nucleation of STS was formulated in Ref. 22, where some K_2_O and B_2_O_3_ was added to the composition, this model was questioned in Ref. 5 where it was noted that the crystal growth morphology in Ref. 22 may rather imply dendritic growth. Dendritic growth in glass-ceramics has e.g. been shown to occur in the case of BTS grown via electrochemically induced nucleation^15^,^16^,^17^,^18^. Similar growth morphologies were observed in STS grown in the same way^19^. Hence, some indicators toward dendritic growth of STS exist while it is not observed during the surface crystallization of samples containing excess SiO_2_ at 970°C^5^. Instead it was proposed that STS is interpenetrated by nm-scale formations of residual glass and the final texture is the fastest way for the crystals to circumvent SiO_2_-barriers which continuously appear in the main direction of growth. SiO_2_ was proposed to form elongated channels permeating the crystallized areas. Similar nm-scale inclusions were also observed after crystallizing a slightly different glass composition where a phase separation was described to occur in the glass before crystallization^23^. Here these inclusions were proven to be amorphous SiO_2_ by TEM-analysis.

In the literature, simulations have shown that the mechanisms of dendritic growth and viscous fingering may occur in the same system by changing the viscosity of the interacting fluids^24^. While it must be noted that these simulations concern the interaction of two liquids, crystal growth morphologies indicating tip splitting and possibly viscous fingering have been observed in glass ceramics^25^,^26^. Multiple growth mechanisms of one phase have been observed within one glass-ceramic sample, e.g. the dendritic and polygon growth of YAG localized to a stria of slightly different chemical composition^27^. Please note that these mechanisms each lead to single crystalline areas of a certain morphology which is in contrast to agglomerations of single crystals such as spherulites^28^ which are not being discussed here.

In contrast to most liquids, glasses have the unique property of passing through a viscosity range of many orders of magnitude during cooling. Hence a crystal phase growing in a glass melt that is cooled below T_g_ (viscosity ≈ 10^13^ dPa s) is surrounded by a matrix spanning an extreme viscosity range during the process which may affect the mechanism of crystal growth, e.g. via diffusion rates. In the case of the BTS glass related to the STS glass discussed in this article, the viscosity at 1445°C (the melting point of BTS) is about 10^2.5^ dPa s^21^.

The Sr_2_TiSi_2_O_8_ and Ba_2_TiSi_2_O_8_ systems form solid solutions with each other as well as with systems in which SiO_2_ is replaced by GeO_2_^29^,^30^,^31^. The resulting fresnoite structures are polar, however, they are not ferroelectric^32^. The latter means they cannot be poled in the presence of an electric field. Fresnoite itself is a polar crystal of the space group P4bm^33^,^34^ and its structure is still frequently analyzed in detail^35^. In order to achieve macroscopic polarity, the crystallization must be carried out in a manner which directly results in the formation of oriented structures, i.e. in an aligned polar axis (crystallographic c-axis). Due to the low relative dielectric constant and the high Curie temperature, piezoelectric fresnoite glass-ceramics are of special interest for high temperature and high frequency applications^36^. It should also be mentioned that the mechanical properties of transparent glass-ceramics containing nm-scale fresnoite crystals have recently been reported^37^.

In this paper, the growth of STS is analyzed; it is shown that STS growth may occur in the form of dendrites as well as probably in the form denoted in the literature as viscous fingering. The conditions for dendritic growth are described and the possible link towards viscous fingering established.

## Results

**Figure 1:**
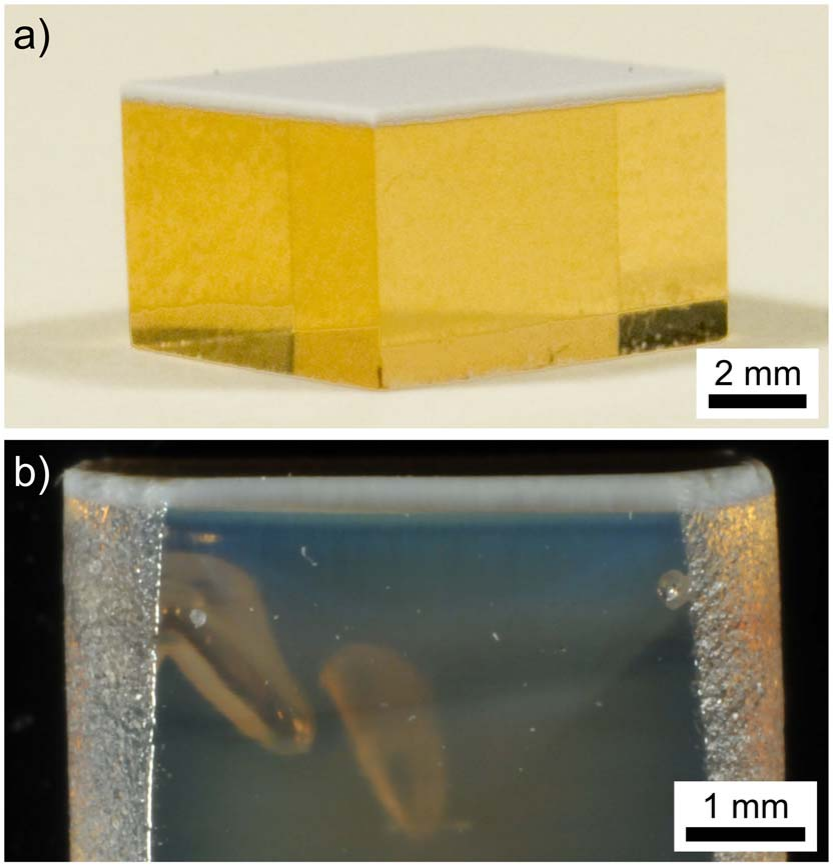
Photographs of polished cross sections. (a) a quenched sample and (b) a quenched sample annealed at 970°C for 2 h after initial cooling.

After quenching and cooling, the samples showed a white layer covering a transparent glass bulk as shown in Figure 1 a). After the second annealing step, the bulk material below the white layer shows the translucency in analogy to that recently described for STS samples annealed for short periods of time^5^,^23^, see Figure 1 b).

**Figure 2:**
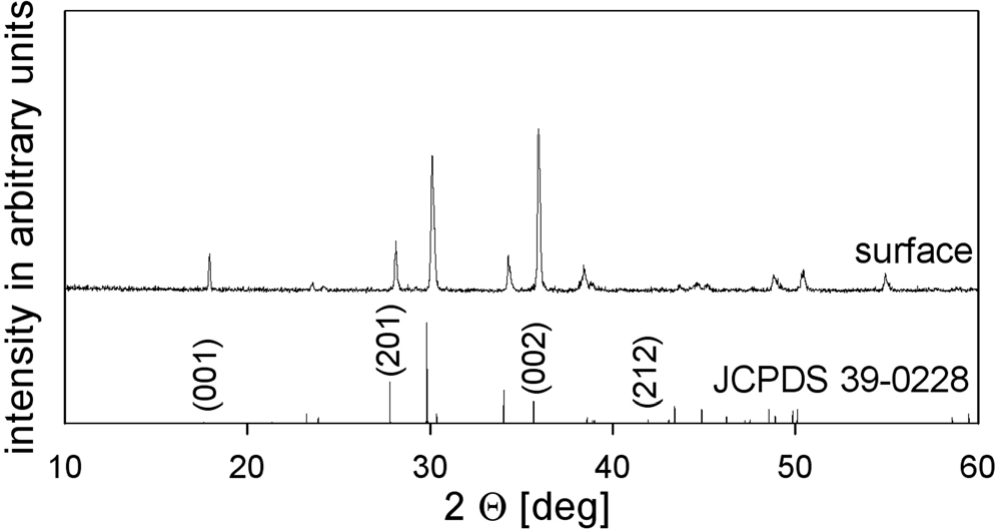
XRD-pattern obtained from the solid surface of a quenched sample along with the theoretical pattern of statistically oriented STS. The (00n)-peaks are of exaggerated intensity in the measured pattern.

An X-ray diffraction (XRD) pattern recorded from the white layer is presented in Figure 2 and shows peaks indicating STS (JCPDS No. 39-0228). The relative intensity of the 00n-peaks is slightly exaggerated indicating a certain degree of orientation preference. However, the orientation preference detected by XRD was much stronger after conventional surface crystallization^5^, i.e. annealing a cooled glass^1^,^2^,^3^,^4^,^5^,^6^,^7^,^8^ without inducing crystallization by pressing with a copper stamp.

**Figure 3:**
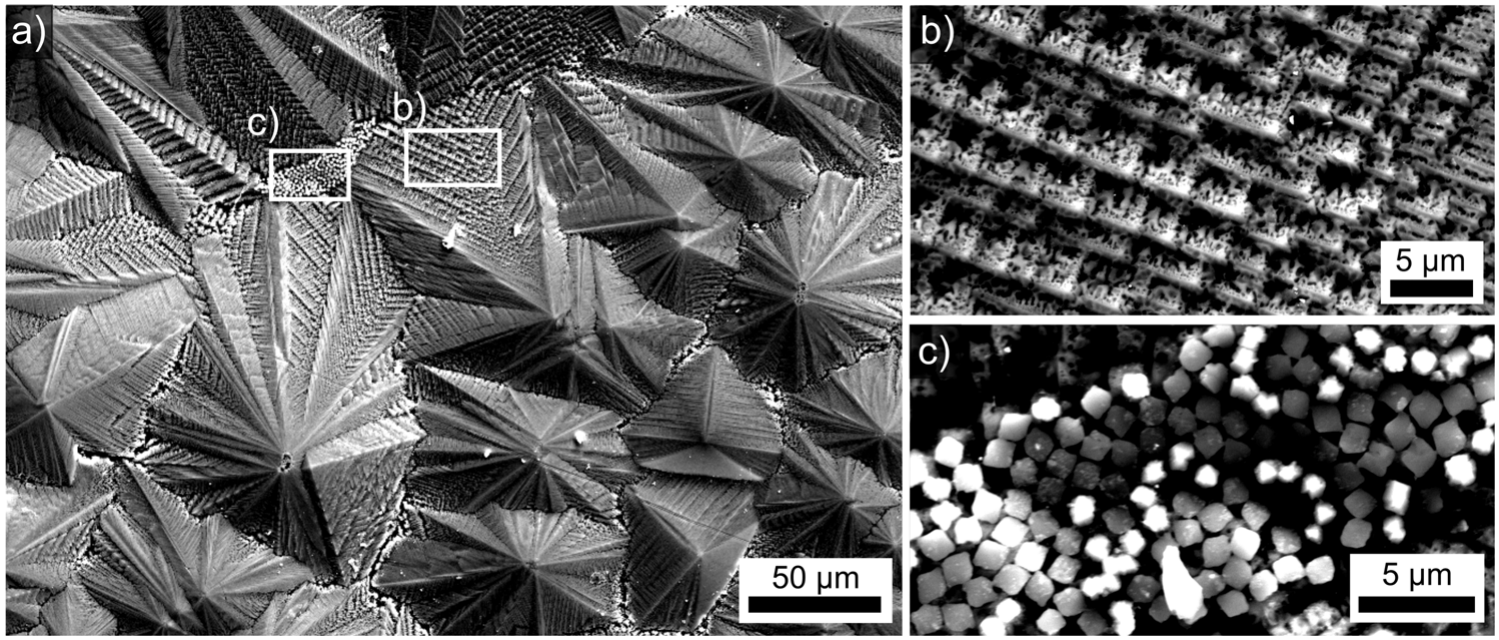
SEM-micrographs of (a) the quenched surface. (b) the morphology of the main growth structures in greater detail, (c) square structures only observed between the main growth structures.

An overview of this quenched surface is presented in Figure 3 a). Large structures with a peak to valley topography of about 20 μm are observed. EBSD-patterns obtained from these structures could be reliably indexed as STS but large EBSD-scans are impossible due to shadowing effects caused by the topography. Figure 3 b) features the detailed morphology of these large structures which is typical for dendritic growth. Figure 3 c) presents a part of the surface where small square structures are frequently observed between the large dendrites. EBSD-patterns obtained from these crystals could be indexed as cubic SrTiO_3_ which matches the chemical information acquired by EDX-analysis using 15 kV.

**Figure 4:**
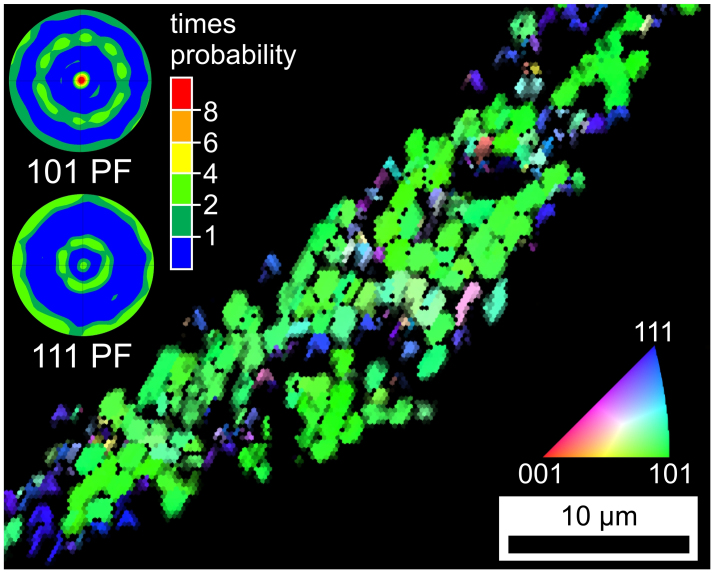
IPF + IQ-map an EBSD-scan performed on the square SrTiO_3_ crystals highlighted in Figure 3 (c). The stereographic projection pole figures of 101- and 111-textures calculated from the scan indicate a double texture in this phase.

The combined Inverse Pole Figure and Image Quality map (IPF + IQ-map) of an EBSD-scan performed on the valley framed in Figure 3 c) is presented in Figure 4. The map only contains data points attributed to SrTiO_3_. The 101-pole figure of a texture calculated from the data set confirms the impression of a 101-orientation gained from the mainly green color in the IPF-map. If the green parts of the IPF-map are ignored, most crystals appear in blue, indicating a 111-orientation. This is backed by the 111-pole figure of the texture also presented in Figure 4, where the central peak is caused by the (111)-planes parallel to the surface while the middle and outer ring are caused by the [111]-planes of the crystals oriented with the [101]-planes parallel to the surface, i.e. the primary orientation.

**Figure 5:**
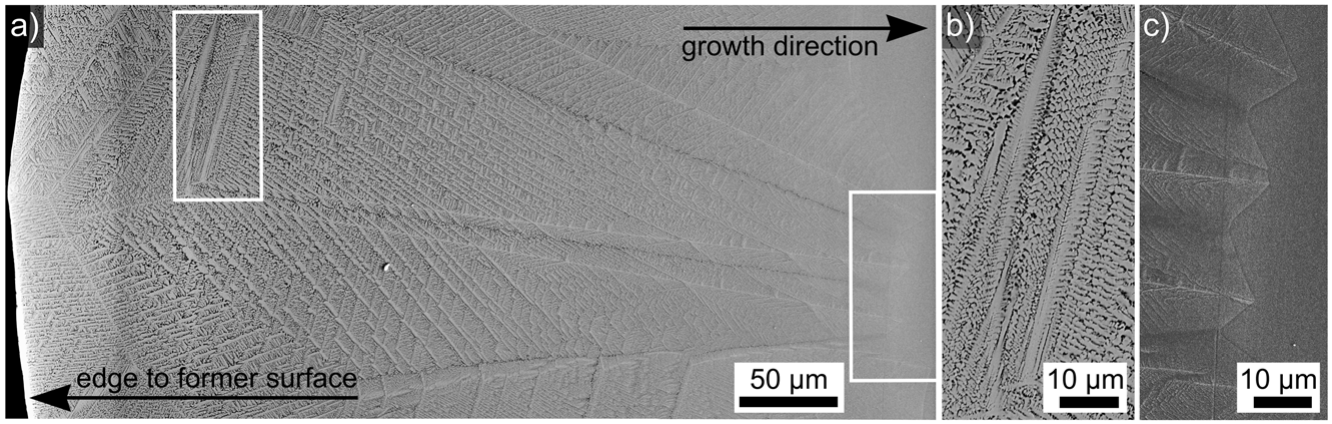
SEM-micrographs of a STS dendrite transecting the entire cross section of the crystallized layer of a quenched sample. The framed areas are presented in detail to illustrate (b) the growth just below the surface, i.e. at high temperatures and (c) the crystallization front after growth was stopped by low temperatures.

Cross sections perpendicular to the surfaces of quenched samples were prepared in order to analyze crystal growth into the bulk. In these cross sections, SrTiO_3_ was never detected using either EBSD or XRD, i.e. SrTiO_3_ most probably only occurs at the surface. Figure 5 a) presents a dendritic growth structure of STS transcending the entire crystallized area. The framed areas are presented in Figure 5 b) and c) in greater detail in order to visualize the growth morphologies at opposite ends of the crystallization process after quenching the sample as described above. As growth proceeds into the volume, the dendritic arms become finer and are more difficult to distinguish from the residual glass matrix (dark). Similar to the controlled crystallization at lower temperatures^5^, chemical differences between the uncrystallized bulk glass and the glass-ceramic layer cannot be detected by EDX, i.e. there is no relevant accumulation of SiO_2_ or impurities at the growth front.

**Figure 6:**
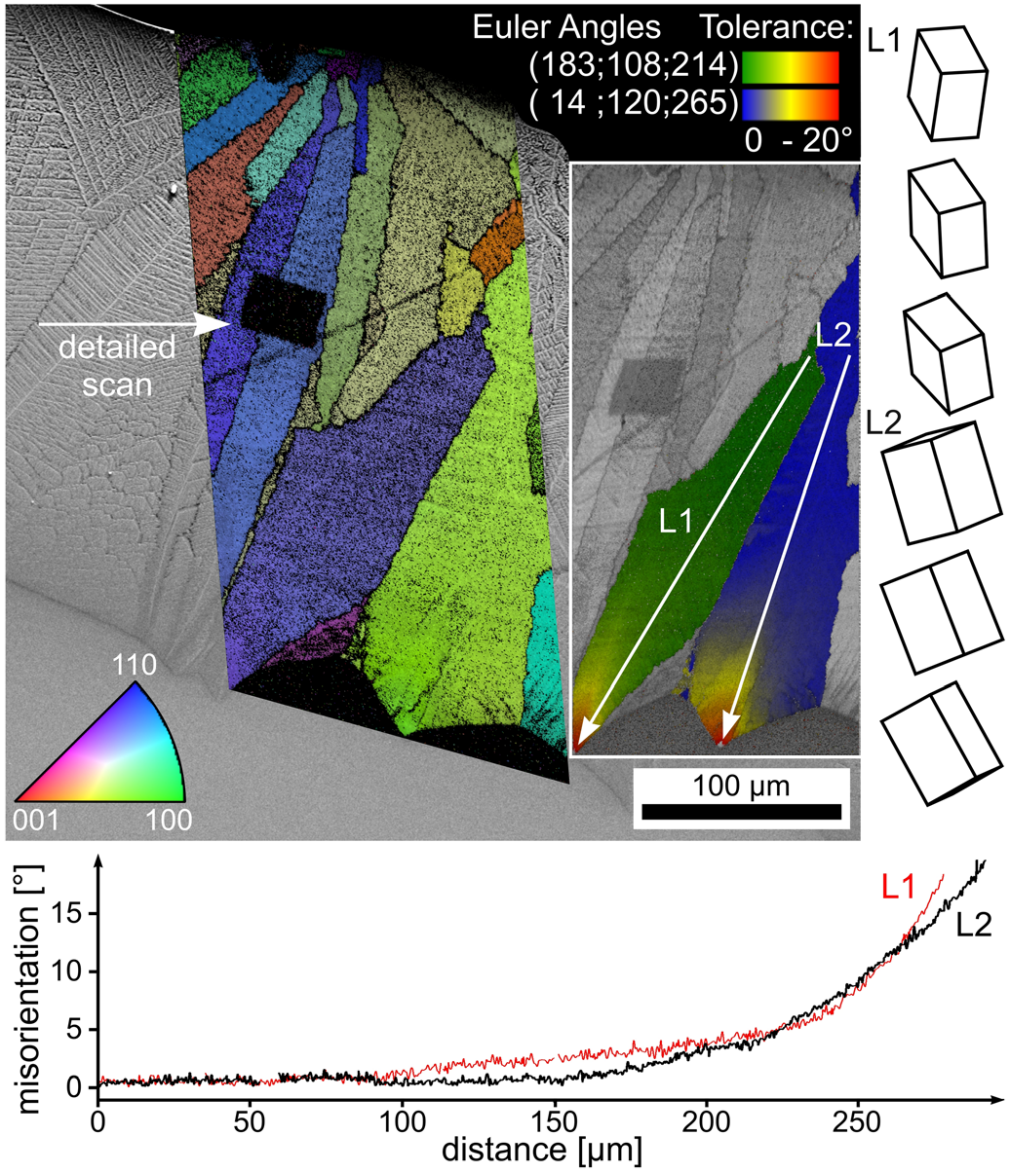
SEM-micrograph of the cross section of a quenched sample superimposed by the IPF + IQ-map of an EBSD-scan. An orientation + IQ-map of the same scan is presented to the right highlighting orientational changes in two specific dendrites. The point-to-origin misorientation profiles presented below indicate an increasing rate of orientation change over distance along the lines L1 and L2.

An SEM-micrograph superimposed by the IPF + IQ-map of an EBSD-scan performed on a similar area is presented in Figure 6. The black area in the map was caused by a lowered EBSD-pattern quality due to a previously performed EBSD-scan of higher detail attempting to resolve the individual dendritic structures. At a first glace it appears as though the crystal orientations within the dendrites of the IPF + IQ-map are homogeneous as expected and dendritic fragmentation^16^,^38^ is not observed. However, the more detailed orientation analysis presented in the orientation + IQ-map (inset) shows a systematic orientation change of up to 20° near the tips of the dendrites. The misorientation profiles along the lines L1 and L2 are presented below and show that the orientation change begins at approximately the same distance from the surface, i.e. the same time after quenching and hence at the same temperature. The changes in the orientation are more pronounced with increasing distance from the surface, which may be explained by the decrease in the crystal growth velocities with decreasing temperature. In any case, the systematic orientation change itself indicates a change in the occurring growth mechanism. The presented unit cells illustrate the orientation at the beginning, middle and end of the line L1 and L2. They indicate that the orientation inside these dendrites changes from the initial orientation at the surface, which is stable for the first 200 μm of growth, towards orientations with their c-axis tilted by about 45° from the surface normal. The same texture has already been described to be stable after growth selection in samples of the same composition after isothermal annealing at 970°C^5^.

**Figure 7:**
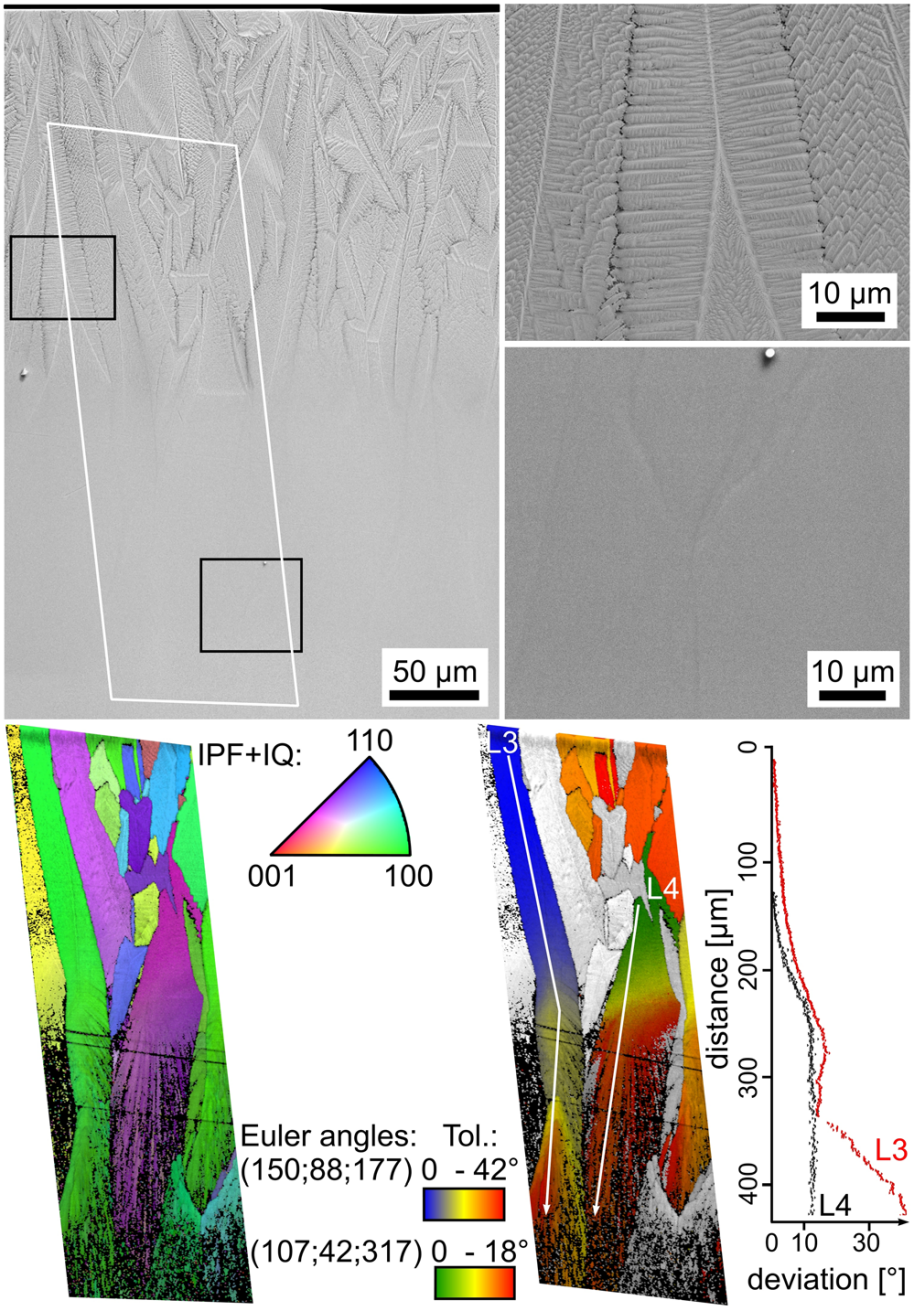
SEM-micrographs of the cross section of a quenched sample subsequently annealed at 970°C for 2 h. The frames highlight dendritic growth near the surface and a low material contrast at what was the end of the primary step of crystal growth. The IPF + IQ-map displays comparably homogeneous orientation and growth near the surface but fraying of areas of homogeneous orientation during the second step of crystal growth. The orientation + IQ-map shows that the orientation remains constant after the initial change.

In order to verify this presumption, quenched samples were placed in a furnace preheated to 970°C and held for 2 h at this temperature for a second annealing step. As bulk nucleation does not occur in this glass, crystal growth should continue where it was stopped during the initial cooling process. SEM-micrographs featuring the cross section of such a sample are presented in Figure 7. The top frame highlights dendritic growth near the surface while the bottom frame shows very little contrast. While this is comparable to SEM-micrographs parallel to the growth direction in samples crystallized at 970°C^5^, a distinct tip characteristic for the crystallization front after primary crystallization (see Figures 5 and 6) is observed. The area framed in white was scanned by EBSD and the resulting IPF + IQ- and orientation maps are presented below. In analogy to Figure 6, the orientations within the dendritic area are very homogeneous during initial growth. The systematic changes of orientation start at a depth comparable to that in Figure 6 and reach a certain deviation which may then be stable, see the lines L3 and L4. In both cases a new, optimal growth direction will be established at some point.

The continuation of the orientations beyond the boundaries of the primary crystallization step confirms that growth is simply resumed during the second annealing step. While the orientation remains comparably stable, the homogeneously oriented areas increasingly fray into the fine grained growth making EBSD-pattern acquisition increasingly difficult. The same process was observed just below the surface of surface crystallized samples^5^.

## Discussion

During the quenching procedure, the copper stamp is in contact to the surface for only a few seconds before it is removed. During this time, the glass at the surface hence passes through a temperature range of maximum nucleation rate. After the stamp is removed, the cooled regions are reheated by the heat capacity of the bulk and passes temperatures of high crystal growth velocities. Hence the temperature/time schedule is not comparable to that applied for nucleation experiments, which is why nucleation plays a minor role in this article.

Nucleation at the surface takes place in contact with the copper stamp during quenching. The obtained nucleation of SrTiO_3_ is not random but occurs with two preferred orientations. However, the probability that the [111]-planes are oriented parallel to the surface, is by far not as high as an orientation of the [101]-planes parallel to the surface. The occurrence of SrTiO_3_ is limited to a surface near region and only STS continues to grow into the bulk.

The results presented above clearly prove that STS grows dendritic at high temperatures, i.e. if the viscosity of the glass matrix is low enough and the growth velocity is high. As the temperature is reduced, the dendrites become finer but still keep the same lattice of the initial dendrite. The increasingly fine structures are contrary to normal isothermal dendrite evolution which tends to coarsen with time in order to reduce the surface energy^38^. However, with decreasing temperature the diffusion rates decrease exponentially and it seems logical that the finer dendrites result from shorter diffusion paths, i.e. growth becomes increasingly diffusion dependent during cooling.

At a certain temperature, the viscosity of the matrix becomes too high to sustain the high diffusion rates necessary for dendritic growth and the mechanism changes to the one described in Ref. 5 where crystals circumvent SiO_2_-diffusion barriers which continuously appear in the main growth direction. As the optimal crystal orientation for this growth mechanism is with the c-axes tilted by about 45° to the main growth direction, the change in the growth mechanism is accompanied by the described systematic orientation change. Finally, the initially homogeneous structures fray into segments of increasingly fine grained crystalline growth highly permeated by nm-scale channels of residual glass.

This later morphology is very similar to the structures observed after 3D-viscous fingering. There is a formal similarity between calculations of viscous fingering and dendritic growth if the viscosity of one fluidic phase is not decisive^39^, e.g. crystals in a glassy matrix because the viscosity of a crystal is infinitely large. Additionally, a circular interface during experiments concerning viscous fingering introduces tip splitting to the process which is also observed in crystallization processes^39^.

On the other hand, the coexistence of viscous fingering and dendritic growth has already been achieved experimentally. After this observation was initially explained by anisotropy^40^, subsequent experiments showed that anisotropy is not essential. Instead the instabilities of dendritic growth may be induced by an increased growth velocity^41^. Hence it appears the growth velocity is the governing parameter and not the matrix viscosity. It has been shown that different growth velocities in a homogeneously viscous matrix may lead to different growth mechanisms.

Unfortunately the counter experiment to test the hypothesis “the growth mechanism depends solely on the viscosity” is probably impossible as it seems impossible to change the matrix viscosity without affecting any other parameter.

For example, air has also been used as a low viscous fluid^42^, so the question arises whether a crystallization front may also be treated as a fluid. It was observed that tips split as soon as the radius of a tip becomes too large for a given surface tension^42^. In the case of STS-crystallization, this would be the point where the SiO_2_-diffusion barrier in the main growth direction becomes too large and secondary growth directions begin to dominate growth^5^.

If correct, these experiments confirm the link between dendritic growth and viscous fingering for the case of a true crystallization process. This was previously only described as a result of numerical simulations and for fluid-fluid interactions.

## Methods

A glass of the batch composition 2SrO**·**TiO_2_**·**2.75SiO_2_ was melted from a mixture of the reagent grade raw materials SrCO_3_, TiO_2_ and SiO_2_ in a Pt-crucible at 1550°C in an inductive furnace for 2 h and stirred for another 2 h at this temperature to homogenize the melt. The glass was then poured on a brass block, quenched for about 3 s with a copper stamp to induce crystallization at the surface and subsequently transferred to a furnace preheated to 760°C which was subsequently turned off, cooling the samples to room temperature with a rate of about 3 K/min. Some of these surface crystallized samples were placed in a furnace preheated to 970°C and then held for 2 h after which the furnace was again turned off to achieve a comparable cooling rate. This procedure is referred to as the second annealing step.

Samples were ground and polished with shrinking grain sizes down to 0.75 μm diamond suspension. A final finish of at least 30 min using colloidal silica was applied for the EBSD-analysis of polished surfaces.

The samples were characterized using a scanning electron microscope Jeol JSM-7001F equipped with an analyzing system EDAX Trident. EBSD-scans were captured and evaluated using a TSL Digiview 3 EBSD-Camera and the software TSL OIM Data Collection 5.31 and TSL OIM Analysis 5.31. In order to achieve a conductive surface, the samples were mounted using an Ag-paste and coated with a thin layer of carbon at about 10^−3^ Pa.

A material file for indexing EBSD-patterns of STS was built based on ICSD-file 59604. The crystalline phase SrTiO_3_ found at the immediate surface was indexed based on the ICDS-file 23076.

## Author Contributions

W.W. and C.R. conceived the experiments. W.W. performed the electron microscopic investigations. M.P. produced the samples. All authors contributed to the discussion and to the writing of the manuscript.
